# Comparative Hepatic Transcriptomic Analysis Reveals Metabolic Regulatory Differences Between Qilian and Oula Sheep

**DOI:** 10.3390/vetsci13050477

**Published:** 2026-05-15

**Authors:** Yaxiong Ren, Qi-Tala An, Xiaohua Du, Xia Liu, Fanhong Gao, Yuan Li, Ying Xu, Liangwei Yao, Wenhao Li

**Affiliations:** 1 College of Veterinary Medicine, Gansu Agricultural University, Lanzhou 730070, China; renyx0724@163.com (Y.R.); anqitala@163.com (Q.-T.A.); gaofh0761@163.com (F.G.); 2 College of Life Science and Technology, Gansu Agricultural University, Lanzhou 730070, China; liux@gsau.edu.cn; 3 College of Animal Science, Xizang Agricultural and Animal Husbandry University, Linzhi 860000, China; quan10242024@icloud.com; 4 Academy of Animal and Veterinary Sciences, Qinghai University, Xining 810016, China; xuy2040@163.com (Y.X.); yaolwe@163.com (L.Y.)

**Keywords:** plateau, lipid, metabolism, PPAR, β-oxidation, validation

## Abstract

Qilian sheep are an important indigenous Tibetan sheep breed adapted to cold and hypoxic environments. To explore hepatic nutrient metabolism in Qilian sheep, this study compared liver transcriptomic profiles between Qilian sheep and Oula sheep raised under similar natural grazing and management conditions. Six 10-month-old ewes from each breed were selected, and three high-quality liver RNA samples from each group were used for transcriptome sequencing. A total of 1640 differentially expressed genes were identified using the thresholds of |log2FoldChange| > 1 and false discovery rate < 0.05, including 922 upregulated and 718 downregulated genes in Qilian sheep compared with Oula sheep. KEGG enrichment analysis showed that these genes were mainly associated with lipid metabolism, amino acid metabolism, the PPAR signaling pathway, fatty acid biosynthesis, and fatty acid β-oxidation. qRT-PCR validation confirmed the differential expression of 11 candidate genes, including *RGN*, *LPGAT1*, *BHMT2*, *SDS*, *GK*, *PC*, *MIOX*, *HMGCS2*, *PNPLA3*, *ACAA2*, and *HADHA*. These results suggest that Qilian sheep and Oula sheep differ in hepatic gene expression patterns related to lipid utilization, amino acid metabolism, and energy regulation, providing candidate genes and pathways for understanding breed-associated hepatic metabolic characteristics.

## Simple Summary

Qilian sheep are a local Tibetan sheep breed living in the high-altitude pastoral areas of the Qinghai–Tibet Plateau. Their ability to survive under cold, hypoxic, and seasonally nutrient-limited conditions may be closely associated with special metabolic regulation. In this study, we compared liver gene expression between Qilian sheep and Oula sheep raised under similar grazing conditions. The results showed that the two breeds differed in genes associated with energy use, lipid utilization, and amino acid metabolism. These findings help explain the biological characteristics of Qilian sheep from the perspective of liver metabolism and may provide useful information for the conservation and utilization of Tibetan sheep genetic resources.

## 1. Introduction

The Qinghai–Tibet Plateau is characterized by high altitude, hypoxia, low temperature, strong ultraviolet radiation, and seasonal forage shortage. These environmental conditions pose major challenges to energy acquisition, nutrient utilization, and the maintenance of metabolic homeostasis in grazing ruminants [1,2,3]. Tibetan sheep are one of the most widely distributed indigenous livestock species on the Qinghai–Tibet Plateau [4]. Through long-term natural selection and artificial breeding, Tibetan sheep have developed strong tolerance to cold and hypoxia and play an important role in maintaining local pastoral production and ecological stability [5,6]. However, the molecular mechanisms regulating nutrient metabolism in different Tibetan sheep breeds remain unclear, especially among breeds raised under similar high-altitude grazing conditions. In this study, “adaptation” mainly refers to physiological and metabolic adjustment to long-term high-altitude grazing conditions, rather than direct evidence of genetic adaptation.

Previous studies have shown that high-altitude animals exhibit distinctive physiological and molecular characteristics in energy metabolism, hypoxia response, substance transport, and stress resistance. Compared with low-altitude sheep, Tibetan sheep differ in rumen fermentation, energy substrate allocation, gluconeogenesis, and lipid metabolism [7,8]. In addition, genomic and proteomic studies have identified candidate genes and pathways related to plateau adaptation and energy metabolism in Tibetan sheep and other high-altitude animals. However, most previous studies have focused on comparisons between high-altitude and low-altitude animals, whereas knowledge of nutrient metabolic differences among different Tibetan sheep breeds living under similar high-altitude conditions remains limited [9].

Natural grazing conditions on the Qinghai–Tibet Plateau are often affected by seasonal changes in forage biomass and nutritional quality. During the cold season or periods of forage shortage, reduced digestible energy and protein intake may cause grazing animals to rely more heavily on effective regulation of lipid mobilization, amino acid catabolism, gluconeogenesis, and energy homeostasis [7,8,9]. Therefore, differences in nutrient metabolism among Tibetan sheep breeds may reflect breed-related physiological strategies for maintaining metabolic balance under alpine grazing conditions.

The liver is an important metabolic organ and plays a central role in carbohydrate, lipid, amino acid, and energy metabolism [10]. In ruminants, volatile fatty acids are produced by rumen fermentation, among which propionate can be transported to the liver and converted into glucose through gluconeogenesis, thereby contributing to the maintenance of energy balance [11,12,13]. Although skeletal muscle and rumen epithelium are also closely related to nutrient utilization, they mainly reflect local energy consumption, growth deposition, or absorption processes. In contrast, the liver integrates nutrient substrates absorbed from the digestive tract and coordinates systemic carbohydrate, lipid, and amino acid metabolism. Therefore, the liver is a suitable tissue for investigating systemic differences in nutrient metabolic regulation among sheep breeds.

Several hepatic metabolic pathways, including lipid metabolism, fatty acid oxidation, amino acid metabolism, gluconeogenesis, and the PPAR signaling pathway, are closely associated with nutrient utilization and energy balance in ruminants [14,15]. Therefore, transcriptomic analysis of liver tissue can help identify candidate genes and pathways involved in breed-related metabolic regulation.

Qilian sheep and Oula sheep are both important indigenous Tibetan sheep breeds distributed in high-altitude regions. Qilian sheep are mainly distributed in the Qilian mountainous area of Qinghai Province and are characterized by strong tolerance to cold, hypoxia, and coarse forage [16,17,18]. Oula sheep are also adapted to alpine environments, but they differ from Qilian sheep in growth rate, body conformation, and meat production performance. In the present study, both Qilian sheep and Oula sheep were sampled from Qilian County, Qinghai Province, which reduced, to some extent, the influence of large-scale environmental differences on gene expression. However, because the experimental animals were maintained under natural grazing conditions rather than strictly controlled feeding conditions, individual differences in feed intake, forage composition, and microenvironment could not be completely excluded.

Based on this background, the present study compared liver transcriptomic profiles between Qilian sheep and Oula sheep. The objective was to identify candidate genes and pathways associated with hepatic nutrient metabolism and to provide molecular clues for understanding breed-related metabolic differences under high-altitude grazing conditions.

## 2. Materials and Methods

### 2.1. Experimental Animals and Sample Collection

A total of 12 healthy 10-month-old female sheep were selected from Qianhu Pasture, Qilian County, Haibei Tibetan Autonomous Prefecture, Qinghai Province, China. The sampling site was located in the Qilian Mountain region of northeastern Qinghai Province, at an altitude of approximately 3200 m. The approximate geographical coordinates were 38.0434° N and 100.5912° E. The sampling area is located in an alpine pastoral region of the Qilian Mountains, characterized by high-altitude mountain terrain, natural grasslands, low annual temperature, large diurnal temperature variation, and seasonal fluctuations in forage biomass and nutritional quality. These topographic and ecological conditions represent typical high-altitude grazing environments on the northeastern Qinghai–Tibet Plateau. Six Oula sheep were assigned to the OL group, with an average body weight of 25.0 ± 3.0 kg, and six Qilian sheep were assigned to the BZ group, with an average body weight of 23.0 ± 3.0 kg.

The selection criteria for the experimental animals included the same sex, the same age, similar body condition, absence of obvious disease or injury, normal feeding behavior, and grazing under the same natural pasture and unified management conditions. All experimental animals were grazed on natural pasture under a unified management system without supplementary feeding throughout the experimental period. Sheep were released for grazing at approximately 07:00 each morning and returned to the pasture enclosure between 18:00 and 19:00 each evening, with free access to forage and water during grazing. This management strategy was used to minimize the influence of feeding conditions and management factors on liver gene expression results.

The body weights of the two groups were compared before sampling using an independent-samples *t*-test. No significant difference was observed between the OL and BZ groups (*p* > 0.05), indicating that the difference in average body weight was unlikely to have a major effect on the transcriptomic comparison. Considering that both breeds were raised under the same grazing conditions and were similar in age, sex, physiological status, and body condition, the observed gene expression differences were considered to mainly reflect breed-related metabolic differences rather than differences caused by body weight or feeding management.

Liver tissue samples were collected immediately after slaughter for transcriptome sequencing and validation experiments. The animals were slaughtered according to routine local commercial slaughter procedures by trained personnel, and exsanguination was performed through carotid artery bleeding. Liver tissue samples were collected immediately after death, rapidly frozen in liquid nitrogen, and stored at −80 °C until RNA extraction. Prior to RNA-seq analysis, the quality of total RNA from each sample was evaluated based on RNA integrity number, purity, and concentration. Three representative samples with relatively high RNA quality were selected from each group for transcriptome sequencing, whereas the remaining samples were used for qRT-PCR validation.

The animal study protocol was approved by the Animal Ethics Committee of Qinghai Academy of Animal Science and Veterinary Medicine (Approval No: 2026-QHMKY-002; date of approval: 5 March 2026). The samples used in this study were obtained from animals slaughtered under an approved project within the same research group. No additional animal experiments, interventions, or treatments were performed specifically for this study.

Because this study was conducted under natural grazing conditions rather than a strictly controlled feeding trial, pasture biomass, forage nutritional composition, actual individual feed intake, temperature, and humidity were not directly measured. These factors were considered as potential limitations when interpreting the transcriptomic results.

### 2.2. Transcriptomics Analysis

#### 2.2.1. Total RNA Extraction, Library Construction and Sequencing

Total RNA was extracted from liver tissue using the Trizol reagent method. Briefly, frozen liver samples were ground in liquid nitrogen, lysed with Trizol reagent, and subjected to phase separation with chloroform. RNA was then precipitated with isopropanol, washed with 75% ethanol, air-dried, and dissolved in RNase-free water. RNA concentration and purity were measured using a NanoDrop 2000 microvolume spectrophotometer (Thermo Fisher Scientific, Waltham, MA, USA), and samples with OD260/280 values between 1.8 and 2.0 were considered acceptable for further analysis. RNA integrity was further verified by agarose gel electrophoresis.

Sequencing libraries were constructed using the Hieff NGS^®^ Ultima Dual-mode RNA Library Prep Kit (Yeasen, 12309ES, Shanghai, China) according to the manufacturer’s instructions. Briefly, mRNA was enriched from total RNA and fragmented into short fragments. First-strand cDNA was synthesized using random primers, followed by second-strand cDNA synthesis, end repair, A-tailing, adapter ligation, PCR amplification, and library quality assessment. Qualified libraries were sequenced on the Illumina NovaSeq 6000 platform (Illumina, San Diego, CA, USA) using a paired-end sequencing strategy.

#### 2.2.2. Sequencing Data Analysis

Raw sequencing reads were first subjected to quality control using fastp software (version 0.18.0). Reads containing adapter sequences, reads with an N base proportion greater than 10%, reads consisting mainly of poly-A sequences, and low-quality reads were removed. (The fastp software was obtained from https://github.com/OpenGene/fastp). The clean reads were then aligned to the sheep reference genome Ovis aries reference genome assembly NCBI_GCF_016772045.2 using HISAT2 (v2.1.0). Gene expression levels were quantified using RSEM, and expression abundance was calculated as transcripts per million (TPM). The RSEM software (version 1.2.19) was obtained from https://deweylab.github.io/RSEM/.

#### 2.2.3. Differential Expression Gene Screening

Differential expression between the BZ and OL groups was analyzed using DESeq2 (v1.24). *p*-values obtained for each gene were adjusted using the Benjamini–Hochberg method to control the false discovery rate. Genes with |log2FoldChange| > 1 and FDR < 0.05 were considered significantly differentially expressed genes. In this study, gene expression changes were described as Qilian sheep relative to Oula sheep. Therefore, genes with positive log2FoldChange values were considered upregulated in Qilian sheep, whereas genes with negative log2FoldChange values were considered downregulated in Qilian sheep.

#### 2.2.4. GO Function and KEGG Pathway Enrichment Analysis of Differentially Expressed Genes

GO functional enrichment analysis was performed by mapping differentially expressed genes to the Gene Ontology database, including biological process, cellular component, and molecular function categories. All genes with GO annotation were used as the background gene set. A hypergeometric test was used for enrichment analysis, and *p*-values were adjusted using the Bonferroni correction method. GO terms with corrected *p*-values < 0.05 were considered significantly enriched.

KEGG pathway enrichment analysis was performed by mapping differentially expressed genes to the Kyoto Encyclopedia of Genes and Genomes pathway database. All genes with KEGG pathway annotation were used as the background gene set, and a hypergeometric test was applied to identify significantly enriched pathways. The *p*-values were corrected for multiple testing, and Q-values, defined as FDR-adjusted *p*-values, were used to evaluate enrichment significance. KEGG pathways with Q-values < 0.05 were considered significantly enriched.

These analyses were used to identify biological functions and metabolic pathways associated with hepatic nutrient metabolism, lipid metabolism, amino acid metabolism, and energy regulation.

#### 2.2.5. Quantitative Real-Time PCR Validation

Eleven differentially expressed genes were verified by qRT-PCR, with β-actin serving as internal reference to correct mRNA expression patterns. Primers were designed by Oligo 6.0 and Primer 5.0 (Table 1). Total RNA was reverse-transcribed into cDNA using a reverse transcription kit according to the manufacturer’s instructions. qRT-PCR parameters were set as follows: pre-denaturation at 95 °C for 30 s; followed by 40 cycles of denaturation at 95 °C for 15 s and annealing/extension at 60 °C for 40 s. A melting curve analysis was then performed at 95 °C for 15 s, 60 °C for 60 s, and 95 °C for 1 s. Each experiment was performed with three biological replicates, and the relative expression level of each target gene was calculated using the 2^−ΔΔCt^ method. Primer specificity was confirmed by melting curve analysis, and the qRT-PCR results were used to validate the expression trends observed in RNA-seq.

## 3. Results

### 3.1. Sequence Alignment Statistics with the Reference Genome

The alignment statistics of clean reads to the reference genome indicated high sequencing quality. The total mapped ratio of all samples ranged from 96.11% to 96.43%, the uniquely mapped ratio ranged from 90.81% to 91.67%, and the multiple mapped ratio ranged from 4.76% to 5.30% (Table 2). These results were higher than the commonly accepted criteria for transcriptome analysis, with total mapped reads greater than 70%, uniquely mapped reads greater than 70%, and multiple mapped reads lower than 10%. The high mapping rate and low multiple-mapping rate indicated that the sequencing data were reliable and suitable for subsequent gene expression quantification, differential expression analysis, and functional enrichment analysis.

### 3.2. Expression Analysis of Liver Tissue Transcriptome Sequencing Data

**Figure 1:**
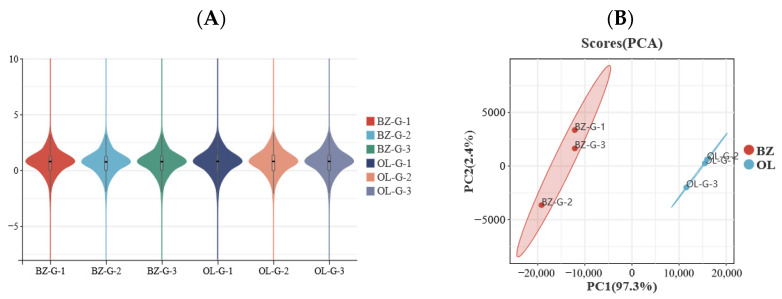
Expression patterns of mRNAs in liver tissue of Qilian sheep and Oula sheep. (**A**) Violin plot of mRNA expression levels; (**B**) principal component analysis of liver transcriptome samples. The percentages on the PCA axes indicate the proportion of variance explained by each principal component. Abbreviations: OL, Oula sheep group; BZ, Qilian sheep group.

Analysis of gene expression distribution revealed generally consistent expression patterns within each group and clear differences between groups. The violin plot showed that the overall distribution of gene expression levels was similar among biological replicates within the same group, indicating good repeatability of the sequencing data (Figure 1A).

Principal component analysis further showed clear separation between the BZ and OL groups (Figure 1B). PC1 explained 97.3% of the total variance and represented the major source of variation between the two breeds, whereas PC2 explained a smaller proportion of the total variance and mainly reflected variation among biological replicates within groups. The BZ and OL samples were clearly separated along PC1, indicating that breed-associated differences were the dominant factor affecting liver transcriptome profiles in this study. The relatively close clustering of samples within each group further supported the consistency of biological replicates and the reliability of subsequent differential expression analysis.

### 3.3. Gene Differential Expression Analysis

**Figure 2:**
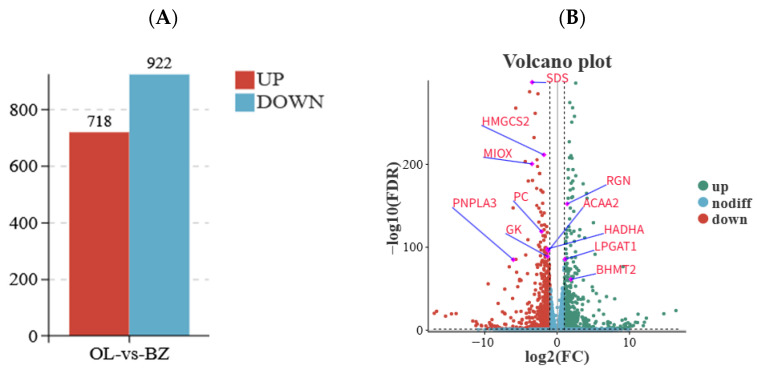
Histogram and volcano plot of differentially expressed genes in liver tissue between Qilian sheep and Oula sheep. (**A**) A total of 1640 significantly differentially expressed genes were identified. Compared with Oula sheep, 922 genes were upregulated and 718 genes were downregulated in Qilian sheep. (**B**) The x-axis represents log2 (fold change), and the y-axis represents −log10 (FDR). Positive log2 (fold change) values indicate higher expression in Qilian sheep, whereas negative values indicate higher expression in Oula sheep. Red and green dots represent significantly differentially expressed genes, whereas blue dots represent genes without significant differential expression. Abbreviations: OL, Oula sheep group; BZ, Qilian sheep group.

Comparative liver transcriptome analysis between Qilian sheep and Oula sheep identified 1640 significantly differentially expressed genes (DEGs). Relative to Oula sheep, 922 genes were upregulated and 718 genes were downregulated in Qilian sheep (Figure 2A). The volcano plot further illustrated the global distribution of these DEGs, with significant genes clearly separated from non-significant genes, highlighting pronounced transcriptomic differences between the two breeds (Figure 2B). This result indicated that the liver transcriptomes of the two Tibetan sheep breeds differed markedly, although the animals were sampled from the same high-altitude grazing region.

Among the DEGs, several genes involved in lipid metabolism, energy homeostasis, and amino acid metabolism showed marked expression changes. In particular, *RGN*, *LPGAT1* and *BHMT2* were significantly upregulated in Qilian sheep, whereas *SDS*, *HMGCS2*, *MIOX*, *PNPLA3*, *PC*, *ACAA2*, *HADHA* and *GK* were significantly downregulated compared with Oula sheep. Functionally, these genes are associated with calcium homeostasis and cellular protection, phospholipid remodeling, one-carbon metabolism, amino acid catabolism, ketogenesis, glucose metabolism, and fatty acid β-oxidation. Therefore, their differential expression suggests that Qilian sheep and Oula sheep may differ not only in individual gene expression levels, but also in hepatic metabolic processes related to lipid utilization, amino acid conversion, gluconeogenesis, and mitochondrial energy metabolism.

### 3.4. GO Functional Analysis of Differentially Expressed Genes

**Figure 3:**
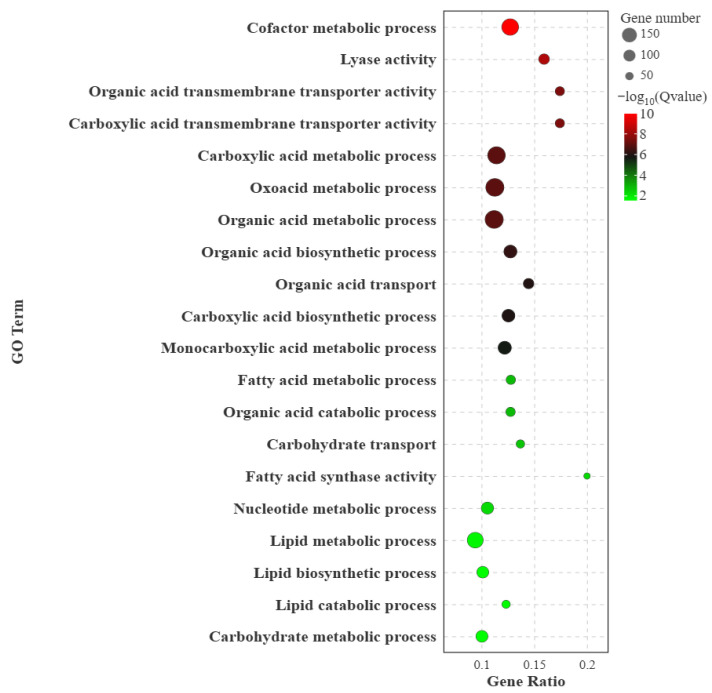
GO enrichment analysis of differentially expressed genes in liver tissue between Qilian sheep and Oula sheep. The bubble plot shows the significantly enriched GO terms of differentially expressed genes identified between Qilian sheep and Oula sheep. The x-axis represents the gene ratio, and the y-axis represents enriched GO terms. The size of each bubble indicates the number of differentially expressed genes enriched in each term, and the color represents the enrichment significance expressed as −log_10_ (Q value). Abbreviations: Gene Ontology, GO.

GO enrichment analysis showed that the DEGs were mainly enriched in biological processes and molecular functions closely related to nutrient metabolism, including cofactor metabolic process, organic acid transmembrane transporter activity, carboxylic acid metabolic process, oxoacid metabolic process, fatty acid metabolic process, lipid metabolic process, lipid biosynthetic process, and amino acid transmembrane transporter activity (Figure 3).

These enriched GO terms were mainly associated with three functional categories. First, terms such as fatty acid metabolic process, lipid metabolic process, and lipid biosynthetic process indicated that lipid turnover and fatty acid metabolism differed between the two breeds. Second, enrichment of organic acid, carboxylic acid, and oxoacid metabolic processes suggested differences in the conversion and utilization of intermediate metabolites involved in energy metabolism. Third, enrichment of amino acid transmembrane transporter activity and organic acid transporter activity indicated that substrate transport may also contribute to hepatic metabolic differences between Qilian sheep and Oula sheep.

Overall, the GO enrichment results suggest that the hepatic transcriptomic differences between the two breeds were closely related to nutrient substrate transport, lipid metabolism, organic acid metabolism, and energy regulation, rather than being limited to isolated changes in individual genes.

### 3.5. KEGG Metabolic Pathway Enrichment Analysis Results

**Figure 4:**
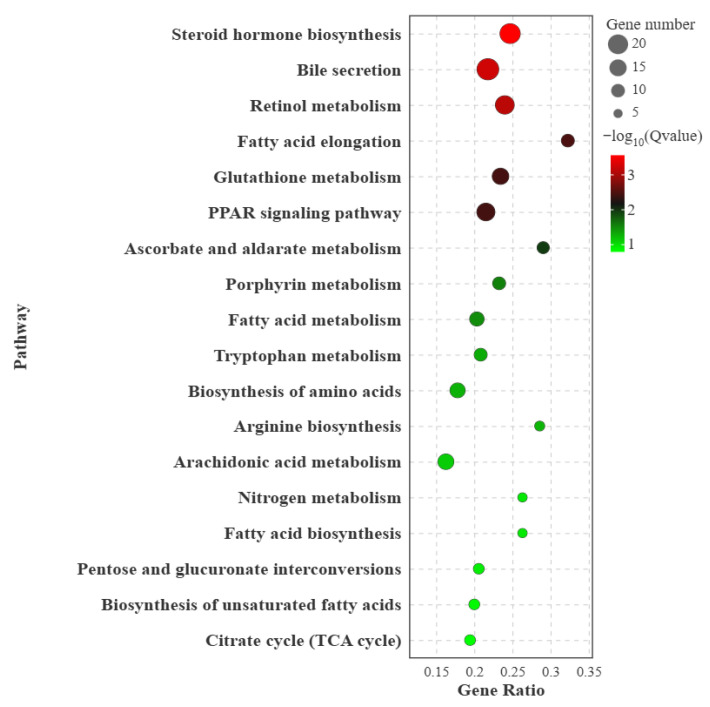
KEGG enrichment analysis of differentially expressed genes in liver tissue between Qilian sheep and Oula sheep. The bubble plot shows the significantly enriched KEGG pathways of DEGs identified between Qilian sheep and Oula sheep. The x-axis represents the gene ratio and the y-axis represents enriched pathways. The size of each bubble indicates the number of differentially expressed genes enriched in each pathway, and the color represents the enrichment significance expressed as −log_10_ (Q value). The enriched pathways were mainly associated with lipid metabolism, amino acid metabolism, bile secretion, retinol metabolism, the PPAR signaling pathway, fatty acid biosynthesis, fatty acid elongation and TCA cycle. Abbreviations: peroxisome proliferator-activated receptor, PPAR; tricarboxylic acid cycle, TCA cycle.

KEGG enrichment analysis revealed a total of 40 enriched pathways between the BZ and OL groups, among which 20 pathways were related to nutrient metabolism (Figure 4). These pathways included steroid hormone biosynthesis, bile secretion, retinol metabolism, fatty acid elongation, glutathione metabolism, PPAR signaling pathway, ascorbate and aldarate metabolism, porphyrin metabolism, fatty acid metabolism, tryptophan metabolism, biosynthesis of amino acids, arginine biosynthesis, arachidonic acid metabolism, carbon metabolism, fatty acid biosynthesis, nitrogen metabolism, pentose and glucuronate interconversions, biosynthesis of unsaturated fatty acids and citrate cycle.

These enriched pathways showed that the metabolic differences between Qilian sheep and Oula sheep were mainly concentrated in lipid metabolism, amino acid metabolism, carbon metabolism, antioxidant metabolism, and vitamin-related metabolism. In particular, enrichment of fatty acid metabolism, fatty acid biosynthesis, fatty acid elongation, fatty acid degradation, and biosynthesis of unsaturated fatty acids indicated that hepatic lipid metabolic remodeling was an important feature distinguishing the two breeds. The enrichment of the PPAR signaling pathway further suggested that transcriptional regulation of lipid utilization and energy balance may be involved in breed-associated hepatic metabolic differences.

In addition, the enrichment of carbon metabolism, the citrate cycle, nitrogen metabolism, tryptophan metabolism, arginine biosynthesis, and biosynthesis of amino acids indicated that amino acid conversion and central energy metabolism also differed between the two breeds. Pathways such as glutathione metabolism, retinol metabolism, ascorbate and aldarate metabolism, and porphyrin metabolism suggested that antioxidant capacity, vitamin metabolism, and cofactor-related metabolism may also participate in maintaining hepatic metabolic homeostasis under high-altitude grazing conditions.

### 3.6. qRT-PCR Validation of Differentially Expressed Genes

**Figure 5:**
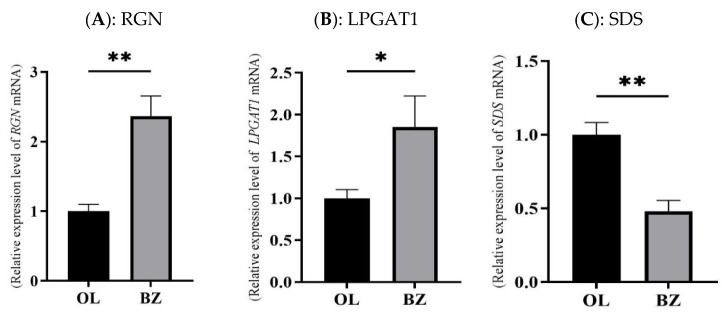

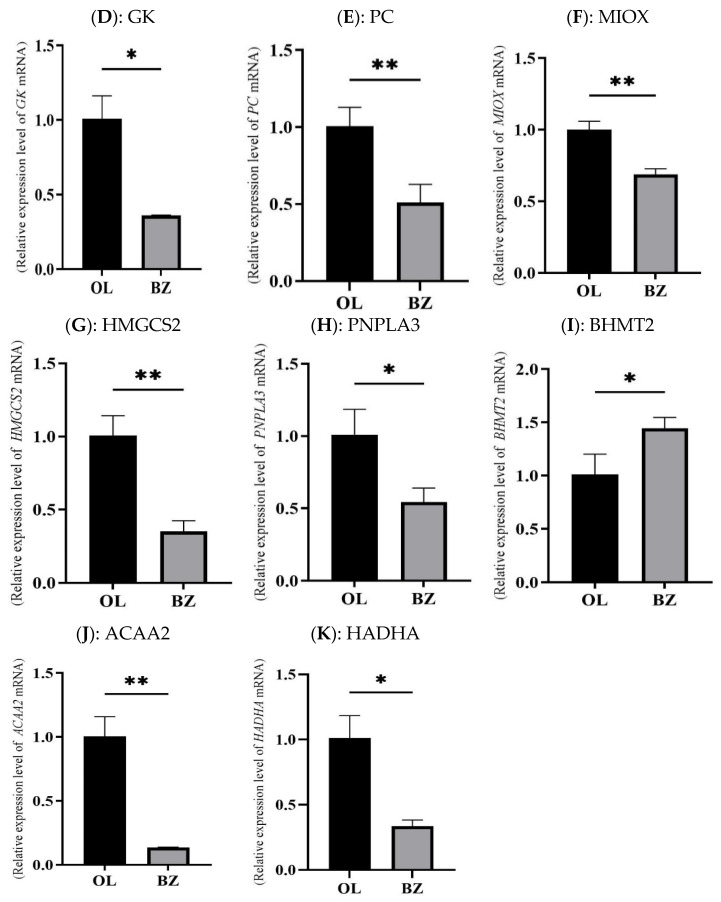
Expression levels of key candidate genes in liver tissue between Qilian sheep and Oula sheep. Panels (**A**–**K**) represent the relative mRNA expression levels of RGN, LPGAT1, SDS, GK, PC, MIOX, HMGCS2, PNPLA3, BHMT2, ACAA2, and HADHA, respectively. Significant differences between groups are indicated as * *p* < 0.05 and ** *p* < 0.01. Abbreviations: OL, Oula sheep group; BZ, Qilian sheep group.

To validate the reliability of the RNA-seq data and further screen key genes involved in nutrient metabolism, a total of 11 representative genes (*RGN*, *LPGAT1*, *SDS*, *GK*, *PC*, *MIOX*, *HMGCS2*, *PNPLA3*, *BHMT2*, *ACAA2*, and *HADHA*) were selected for qRT-PCR validation based on significant differential expression (*p* < 0.05), GO and KEGG enrichment results, and relevant functional annotations (Figure 5).

The qRT-PCR results showed that the mRNA expression levels of *RGN* were highly significantly higher in Qilian sheep than in Oula sheep (*p* < 0.01), while the mRNA expression levels of *LPGAT1* and *BHMT2* were significantly higher in Qilian sheep than in Oula sheep *(p* < 0.05). In contrast, the mRNA expression levels of *SDS*, *PC*, *MIOX*, *HMGCS2*, and *ACAA2* were highly significantly higher in Oula sheep than in Qilian sheep (*p* < 0.01), whereas the mRNA expression levels of *GK*, *PNPLA3*, and *HADHA* were significantly higher in Oula sheep than in Qilian sheep (*p* < 0.05).

The expression trends of these 11 genes were generally consistent with the RNA-seq results, supporting the reliability of the transcriptome sequencing data. From a functional perspective, the upregulation of *RGN*, *LPGAT1* and *BHMT2* in Qilian sheep may be related to cellular homeostasis, membrane lipid remodeling and methyl-group metabolism, whereas the higher expression of *SDS*, *GK*, *PC*, *MIOX*, *HMGCS2*, *PNPLA3*, *ACAA2* and *HADHA* in Oula sheep suggests relatively active amino acid catabolism, glucose metabolism, ketone body metabolism, triglyceride turnover and fatty acid oxidation. These results further indicate that the two breeds may have different hepatic metabolic regulation patterns under similar grazing conditions.

## 4. Discussion

### 4.1. Main Findings and Interpretation of the Study Design

The main finding of this study was that Qilian sheep and Oula sheep showed distinct hepatic transcriptomic profiles under the same natural grazing conditions [16]. Principal component analysis showed clear separation between the two breeds, and differential expression analysis identified 1640 differentially expressed genes, including 922 upregulated and 718 downregulated genes in Qilian sheep compared with Oula sheep. Differential expression and enrichment analyses also identified a series of genes and pathways associated with lipid metabolism, amino acid metabolism, organic acid metabolism, and energy regulation. These results support the hypothesis that different Tibetan sheep breeds living under similar high-altitude conditions may still differ in the expression of genes related to hepatic nutrient metabolism.

Oula sheep were used as the control group because they are also an indigenous Tibetan sheep breed adapted to high-altitude environments, but they differ from Qilian sheep in genetic background, body conformation, growth performance, and meat production traits. Compared with using low-altitude sheep as controls, using Oula sheep reduced the influence of large-scale environmental differences such as altitude, hypoxia, and temperature, allowing a more focused comparison of hepatic metabolic differences between the two Tibetan sheep breeds.

All experimental animals were 10-month-old ewes sampled from Qianhu Pasture in Qilian County, Qinghai Province, and were raised under the same natural grazing and unified management conditions. Therefore, the study design reduced the effects of age, sex, sampling region, and major feeding management factors on liver gene expression. However, because the animals were maintained under natural grazing rather than strictly controlled feeding conditions, individual variation in actual feed intake, grazing behavior, forage composition, and short-term physiological status could not be completely eliminated. Therefore, the results should be interpreted as breed-related transcriptomic differences observed under relatively consistent natural grazing conditions, rather than as purely genetic effects independent of environmental influence.

The liver is a central organ for lipid metabolism, amino acid metabolism, gluconeogenesis, and energy homeostasis in ruminants [17]. Therefore, the transcriptomic differences observed in liver tissue provide useful molecular evidence for understanding how Qilian sheep and Oula sheep may differ in nutrient utilization and metabolic regulation. This is the main contribution of the present study to molecular genetics: it identified candidate genes and pathways that may be associated with breed-related hepatic metabolic differentiation within Tibetan sheep.

### 4.2. Gene Ontology Enrichment Suggests Differences in Nutrient Substrate Utilization

Gene Ontology enrichment analysis showed that differentially expressed genes were mainly enriched in lipid metabolic process, fatty acid metabolic process, organic acid metabolic process, carboxylic acid metabolic process, oxoacid metabolic process, and several transmembrane transporter activities. These enriched terms are directly related to hepatic nutrient substrate conversion, metabolite transport, and energy supply, indicating that the two breeds differ not only in individual gene expression but also in broader functional categories related to nutrient metabolism.

The enrichment of lipid- and fatty acid-related biological processes suggests that hepatic lipid turnover is an important component of the metabolic differences between Qilian sheep and Oula sheep. In grazing ruminants, especially those living in cold and high-altitude environments, lipid mobilization and fatty acid utilization are important for maintaining energy balance when forage availability or nutrient quality fluctuates [18]. Therefore, the Gene Ontology results suggest that the two breeds may regulate lipid-derived energy supply differently under the same grazing environment.

Several transporter-related Gene Ontology terms were also enriched, including organic acid transmembrane transporter activity, carboxylic acid transmembrane transporter activity, monocarboxylic acid transmembrane transporter activity, and amino acid transmembrane transporter activity. These results indicate that breed-related differences may involve not only intracellular metabolic reactions but also the uptake, exchange, and export of nutrient substrates. This is biologically relevant because hepatic metabolism depends on the coordinated transport and conversion of amino acids, organic acids, and lipid-related metabolites.

### 4.3. KEGG Enrichment Highlights Lipid Metabolism and Energy Regulation

KEGG enrichment analysis further showed that the differentially expressed genes were enriched in several nutrient metabolism-related pathways, including fatty acid metabolism, fatty acid biosynthesis, fatty acid elongation, fatty acid degradation, steroid hormone biosynthesis, bile secretion, retinol metabolism, glutathione metabolism, tryptophan metabolism, arginine biosynthesis, nitrogen metabolism, carbon metabolism, and the citrate cycle. These pathways indicate that hepatic transcriptomic differences between Qilian sheep and Oula sheep involve coordinated changes in lipid metabolism, amino acid metabolism, antioxidant metabolism, and central energy metabolism.

Among these pathways, the enrichment of fatty acid metabolism, fatty acid biosynthesis, fatty acid degradation, and fatty acid elongation indicates that the two breeds differ in hepatic lipid conversion. Fatty acids can be used as energy substrates, while fatty acid synthesis and degradation jointly influence lipid deposition, lipid mobilization, and energy supply [18]. Therefore, the simultaneous enrichment of multiple fatty acid-related pathways suggests that the difference between Qilian sheep and Oula sheep is not limited to a single metabolic reaction, but may involve the balance among lipid remodeling, lipid utilization, and energy conversion.

The peroxisome proliferator-activated receptor signaling pathway was also enriched [19,20,21]. This pathway is closely associated with fatty acid transport, fatty acid β-oxidation, lipid storage, and energy homeostasis. The enrichment of this pathway provides a pathway-level explanation for the Gene Ontology results related to lipid and fatty acid metabolism and suggests that peroxisome proliferator-activated receptor-mediated regulation may be an important component of hepatic metabolic differentiation between Qilian sheep and Oula sheep.

Similar findings have been reported in other sheep transcriptome studies. For example, liver transcriptome comparisons between Tibetan sheep and Hu sheep revealed differences in genes and pathways related to hepatic fat metabolism. In addition, hepatic transcriptome analyses of sheep with different fatty acid traits also identified genes and pathways involved in fatty acid metabolism, including lipid metabolism-related signaling pathways. These studies support the view that the liver transcriptome is closely associated with lipid metabolism and production-related traits in sheep. Compared with previous studies that mainly focused on breeds from different ecological backgrounds or individuals with different fatty acid phenotypes, the present study focused on two Tibetan sheep breeds sampled from the same high-altitude region, thereby providing more specific information on breed-related hepatic metabolic differentiation within Tibetan sheep populations.

### 4.4. Candidate Genes Provide Molecular Clues for Hepatic Metabolic Differences

Transcriptome sequencing and qRT-PCR results consistently showed that RGN, LPGAT1, and BHMT2 were significantly upregulated in Qilian sheep, whereas SDS, GK, PC, MIOX, HMGCS2, PNPLA3, ACAA2, and HADHA were significantly downregulated. The consistency between RNA-seq and qRT-PCR supports the reliability of the transcriptomic results and indicates that these genes can be considered candidate genes associated with hepatic nutrient metabolic differences between Qilian sheep and Oula sheep.

RGN is related to intracellular calcium homeostasis and cellular metabolic regulation [22,23]; LPGAT1 participates in phospholipid remodeling and membrane lipid homeostasis [24,25]; and BHMT2 is involved in methyl-group metabolism and homocysteine conversion [26]. The upregulation of these genes in Qilian sheep suggests that cellular homeostasis, membrane lipid remodeling, and one-carbon metabolism may participate in the hepatic metabolic characteristics of this breed. However, because stress markers, calcium flux, mitochondrial function, and metabolite levels were not directly measured in this study, these interpretations should be regarded as transcriptome-based hypotheses rather than confirmed mechanisms.

Among the downregulated genes, SDS is involved in serine catabolism and links amino acid metabolism with pyruvate production; GK and PC are associated with glucose metabolism and gluconeogenesis; MIOX participates in inositol metabolism; HMGCS2 is involved in ketogenesis [27]; PNPLA3 participates in triglyceride turnover and lipid droplet remodeling [28,29,30]; and ACAA2 and HADHA are involved in fatty acid β-oxidation [31]. The lower expression of these genes in Qilian sheep, together with the enrichment of fatty acid metabolism and the peroxisome proliferator-activated receptor signaling pathway, suggests that the two breeds may differ in the balance among amino acid catabolism, gluconeogenesis, ketone body production, lipid droplet turnover, and fatty acid oxidation.

Therefore, the candidate genes identified in this study do not directly prove causal mechanisms but provide molecular clues for future functional validation. In particular, RGN, LPGAT1, BHMT2, HMGCS2, PNPLA3, ACAA2, and HADHA may be prioritized in subsequent studies because their known functions are related to cellular homeostasis, lipid remodeling, fatty acid metabolism, and energy regulation.

### 4.5. Possible Biological Explanation for Breed-Related Metabolic Differences

Although Qilian sheep and Oula sheep both belong to Tibetan sheep and live in high-altitude environments, their long-term breeding history, production orientation, and local selection pressures may have shaped different metabolic regulatory patterns. Qilian sheep are generally considered to have strong tolerance to cold, hypoxia, and coarse forage, whereas Oula sheep show relatively better growth performance and meat production potential. These differences in ecological adaptation and production traits may help explain why the two breeds showed different hepatic gene expression patterns related to lipid metabolism, amino acid metabolism, and energy regulation.

From a biological perspective, Qilian sheep may need to maintain relatively stable nutrient utilization regulation under long-term alpine grazing conditions, where forage supply and nutritional quality often fluctuate seasonally. In contrast, Oula sheep may have been selected more strongly for growth and meat production traits, which may be associated with different hepatic regulation of energy storage, amino acid utilization, and lipid turnover. Therefore, the expression differences observed in this study may reflect different physiological priorities between the two breeds: Qilian sheep may be more oriented toward maintaining metabolic homeostasis under harsh grazing conditions, whereas Oula sheep may show metabolic activity more closely related to growth or deposition.

However, this interpretation remains hypothesis-generating. Because this study did not directly measure feed intake, body composition, blood metabolites, sex hormone levels, or liver enzyme activities, the proposed metabolic strategies require further validation. Future studies should combine transcriptomics with controlled feeding trials, metabolomics, proteomics, enzyme activity assays, and production trait measurements to clarify how these gene expression differences are translated into physiological and productive phenotypes.

### 4.6. Applicability to Ewes and Future Research Directions

All animals used in this study were 10-month-old ewes. This design reduced sex- and age-related variation within the experiment, but it also means that the findings should be applied primarily to young female sheep under similar grazing conditions. Because sex hormones can influence liver lipid metabolism, amino acid metabolism, and energy homeostasis, whether the same transcriptomic patterns occur in rams, castrated males, or sheep at other physiological stages remains unknown.

Future studies should include animals of different sexes, developmental stages, and physiological states to determine whether the candidate genes and pathways identified here are specific to ewes or represent more general breed-related metabolic features. In addition, larger sample sizes, actual feed intake monitoring, controlled feeding trials, metabolomics, proteomics, enzyme activity assays, and functional validation are needed to clarify the relative contributions of breed, sex, nutrition, and environment to hepatic nutrient metabolism in Tibetan sheep.

## 5. Conclusions

This study demonstrated that Qilian sheep and Oula sheep exhibit distinct hepatic transcriptomic patterns related to nutrient metabolism under similar high-altitude grazing conditions. A total of 1640 differentially expressed genes were identified, and functional enrichment analysis showed that these genes were mainly involved in lipid metabolism, fatty acid metabolism, amino acid metabolism, organic acid metabolism, PPAR signaling, fatty acid synthesis, fatty acid β-oxidation, and energy regulation. qRT-PCR validation confirmed the differential expression of 11 candidate genes, including *RGN*, *LPGAT1*, *BHMT2*, *SDS*, *GK*, *PC*, *MIOX*, *HMGCS2*, *PNPLA3*, *ACAA2*, and *HADHA*.

These findings suggest that the hepatic nutrient metabolism differences between Qilian sheep and Oula sheep are not limited to individual gene expression changes, but may reflect coordinated regulation of lipid turnover, amino acid conversion, substrate transport, and metabolic homeostasis. Because this study was based on transcriptomic association analysis, the identified genes and pathways should be regarded as candidate molecular indicators and testable hypotheses rather than direct evidence of causal mechanisms.

From a practical livestock perspective, these results provide preliminary molecular information for future evaluation of nutrient utilization efficiency, metabolic adaptability, and breed resource conservation in Tibetan sheep. Future studies should expand the sample size, monitor actual feed intake and pasture nutrient composition, and integrate controlled feeding trials, metabolomics, proteomics, enzyme activity assays, and functional experiments to clarify the biological roles of these candidate genes and their relationships with production traits and plateau adaptability.

## Figures and Tables

**Table 1:** Primer Information.

Genes	Primer	Sequence (5′-3′)	Primer Length/bp	Melting Temperature/°C
*β-actin*	Actin-F	CTTCCAGCCTTCCTTCCTGG	20	58.5
	Actin-R	GCCAGGGCAGTGATCTCTTT	20	57.8
*SDS*	SDS-F	CTCAGACCAGGAAGCTGTGG	20	58.4
	SDS-R	GACCACAACAGAGGACAGGG	20	58.3
*GK*	GK-F	AGAGGGATCATCTGTGGGCT	20	58.2
	GK-R	ATTCCACAGTCGCGGTTCAT	20	57.0
*PC*	PC-F	TAGAGAACTGGGGAGGAGCC	20	58.7
	PC-R	GGGTAGTTGGTGTAGCCCAC	20	58.4
*MIOX*	MIOX-F	GAAACTACACGTCTGGCCCA	20	57.6
	MIOX-R	ATAGGAGAAGCCCCCGAACT	20	58.1
*HMGCS2*	HMGCS2-F	GGAACCCACATGGAGAACGT	20	57.7
	HMGCS2-R	ATTTGTCCAGGGCCCGTAAG	20	57.8
*PNPLA3*	PNPLA3-F	TCAAGGATACGTAGACGCGC	20	57.3
	PNPLA3-R	ACACTCGGAAAAGGCTCCAG	20	57.6
*ACAA2*	ACAA2-F	TTCTGATGGTGCTGGAGCTG	20	57.7
	ACAA2-R	TGATAGCAGGGACAGGACCA	20	58.0
*HADHA*	HADHA-F	GGGGTTTTGAAAAGGCCGAC	20	57.5
	HADHA-R	GCAGATGTGTTGCTGGCAAA	20	56.8
*BHMT2*	BHMT2-F	TGTAGAAGCTGTGTGGGCTG	20	57.6
	BHMT2-R	TCCGCAGCTTCACATCACAT	20	57.0
*LPGAT1*	LPGAT1-F	ATGGTGGCTTCTTGGGGATG	20	58.0
	LPGAT1-R	CCTTTGTCCTGGAGGCACAT	20	57.8
*RGN*	RGN-F	GTGGTGAGTCTCCAGTGTGG	20	58.3
	RGN-R	GAGAGAATCCCACCGGCAAA	20	57.8

**Table 2:** Statistical Results of Reference Genome Alignment.

Sample	Total	Unmapped (%)	Unique_Mapped (%)	Multiple_Mapped (%)	Total_Mapped (%)
BZ-G-1	50,288,644	1,958,430 (3.89%)	45,783,196 (91.04%)	2,547,018 (5.06%)	48,330,214 (96.11%)
BZ-G-2	49,386,340	1,899,764 (3.85%)	44,874,922 (90.87%)	2,611,654 (5.29%)	47,486,576 (96.15%)
BZ-G-3	52,522,332	2,043,090 (3.89%)	47,695,016 (90.81%)	2,784,226 (5.30%)	50,479,242 (96.11%)
OL-G-1	46,391,558	1,668,245 (3.60%)	42,478,173 (91.56%)	2,245,140 (4.84%)	44,723,313 (96.40%)
OL-G-2	48,037,442	1,715,357 (3.57%)	44,033,777 (91.67%)	2,288,308 (4.76%)	46,322,085 (96.43%)
OL-G-3	46,677,004	1,775,553 (3.80%)	42,668,154 (91.41%)	2,233,297 (4.78%)	44,901,451 (96.20%)

## Data Availability

The original contributions presented in this study are included in the article. Further inquiries can be directed to the corresponding authors.
